# The complete chloroplast genome of an endangered orchid *Paphiopedilum spicerianum*

**DOI:** 10.1080/23802359.2020.1830727

**Published:** 2020-10-21

**Authors:** Li-Ping Ge, Lu Tang, Lu Li, Yan Luo

**Affiliations:** a College of Forestry, Shanxi Agricultural University, Jinzhong, China; b Gardening and Horticulture Department, Xishuangbanna Tropical Botanical Garden, Chinese Academy of Sciences, Mengla, China; c Department of Biodiversity Conservation, Yunnan Academy of Biodiversity, Southwest Forestry University, Kunming, China; d Gardening and Horticulture Department, Core Botanical Gardens, Chinese Academy of Sciences, Mengla, China

**Keywords:** *Paphiopedilum spicerianum*, chloroplast genome, Orchidaceae, phylogenomics

## Abstract

*Paphiopedilum spicerianum* (H. G. Reichenbach) Pfitzer is an endangered and threatened orchid species in China. The genetic and molecular data of this orchid species is deficient. With the aim to identify appropriate chloroplast markers for the use in a conservation biology study, the complete chloroplast genome of *P. spicerianum* was reported. We found that the chloroplast genome of *P. spicerianum* is 157,292 bp in length, containing a large single-copy region of 87,252 bp, a small single-copy region of 1828 bp, and a pair of inverted repeats of 34,106 bp. Genome annotation predicted 105 unique genes, including 71 protein-coding genes, 30 tRNAs, and four rRNAs. Fourteen genes contained one intron and two genes (*clp*P and *ycf*3) had two introns. The GC content of the *P. spicerianum* was 35.8%. Phylogenetic analysis indicated *P. spicerianum* was closely related to *P. purpuratum*.

*Paphiopedilum spicerianum* (H. G. Reichenbach) Pfitzer is an endangered orchid with significant ornamental values. It is restricted to eastern and northeastern Myanmar, Thailand, and southwestern China. In China, it is known from only one site in Yunnan Province (Ye and Luo 2006). Due to habitat loss and limited populations, *P. spicerianum* is listed as one of the wild plants with extremely small population by State Forestry Administration of China (Ma et al. 2013). With the popularity of Next-Seq technology, coupled with the moderate length and evolution rate of chloroplast genome, chloroplast genome has been gradually used for species evolution analysis, and can also be used for the establishment of conservative strategies for endangered plant.

The fresh leaves of *P. spicerianum* were collected from orchid nursery of Xishuangbanna Tropical Botanical Garden, Chinese Academy of Sciences, Yunnan Province, China (21°54′N, 101°46′E; voucher specimen: LY0154, deposited in Xishuangbanna Tropical Botanical Garden Herbarium, HITBC). Total genomic DNA from leaf was extracted by using Tiangen DNA kit (TIANGEN, Beijing, China), and sequenced by the Illumina Hiseq 2500 sequencing platform (Illumina, Foster City, CA) at Personal Biotechnology Co., Ltd. (Shanghai, China). The total 3.0 G data of high-quality reads were trimmed, and then used to assemble the chloroplast genome using a toolkit named GetOrganelle (Jin et al. 2018). The assembled chloroplast genome was annotated by the web server GeSeq (https://chlorobox.mpimp-golm.mpg.de/geseq.html, Tillich et al. 2017). The tRNA genes were verified by tRNAscan-SE v2.0.3 (Lowe and Chan 2016). The annotated complete chloroplast genome of *P. spicerianum* was deposited in GenBank with accession number MT683624.

The complete chloroplast genome of *P. spicerianum* was 1,57,292 bp in length, which presented a typical quadripartite structure, and consists of a large single-copy region (LSC, 87,252 bp), a small single-copy region (SSC, 1828 bp), and two inverted repeat regions (IRs, 34,106 bp). The total GC content of chloroplast genome was 35.8%, whereas the corresponding values of LSC, SSC, and IR regions are 33.2%, 23.8%, and 39.5%, respectively*. Paphiopedilum spicerianum* chloroplast genome encoded 105 unique genes, including 71 protein-coding genes, 30 tRNA genes, and four rRNA genes. Fourteen genes had a single intron while the *clp*P and *ycf*3 genes had two introns.

To further investigate its phylogenetic position, the complete chloroplast genomes from 10 species of Cypripedioideae, and one species from Orchidoideae and one species from Epidendroideae as outgroup, were selected to reconstruct phylogenetic tree. Sixty-five protein-coding genes exported from Geneious Primer 2020 (Biomatters, Auckland, New Zealand) were aligned with MAFFT (Katoh and Standley 2013) and Mauve (Darling et al. 2004). A maximum-likelihood (ML) analysis of the plastome data and Best-fit model TVM + F+I + G4 were performed using IQ-TREE-2.0.5 with 1000 bootstrap replicates (Minh et al. 2020). Phylogenetic analysis showed that *P. spicerianum* was closely related to *P. purpuratum* within Cypripedioideae with strong support, and the taxa of *Paphiopedilum* formed a well-supported clade with *Phragmipedium longifolium* (Figure 1).

**Figure 1.**
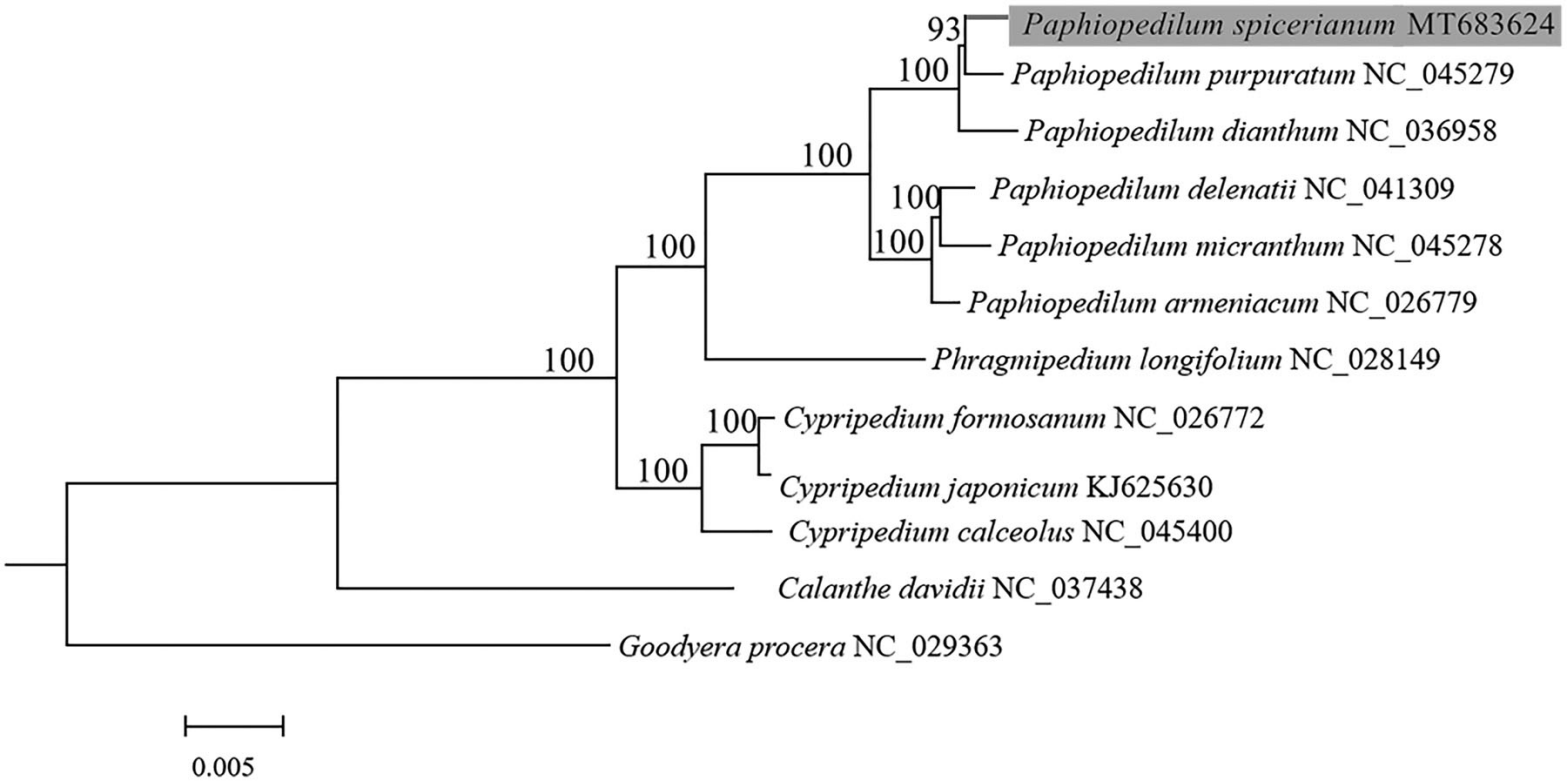
Phylogenetic position of *Paphiopedilum spicerianum* (gray box) inferred by the maximum-likelihood (ML) analysis based on 65 protein-coding genes from chloroplast genome using 1000 bootstrap replicates. The number at each node indicates bootstrap value and the scale is substitution per site.

## Data Availability

The genomic sequence data that support the findings of this study have been deposited in GenBank with accession no. MT683624 (https://www.ncbi.nlm.nih.gov/).
